# Ontario Veterinary Medical Association

**Published:** 1891-03

**Authors:** Allen Z. Keelor

**Affiliations:** Secretary


*Ontario Veterinary Medical Association.*—The Ontario Veterinary Medical Society, which is connected with the Ontario Veterinary College, Toronto, Canada, meets semi-weekly in the College Hall. It has for its aim, the discussion of various subjects, in the way of Essays and Communications, furnished by its members, who consist of the Graduating Class, and in so doing, improving its members, in subjects pertaining to their profession.

The office bearers for 1890-91 are: President, Prof. A. Smith, F.R.C.V.S., Ed.; Vice-Presidents, Prof. J. Thorburn, M. D., Ed., Prof. J. Duncan, M. D., V. S., Prof. A. King, M. D., V. S.; Secretary, A. Z. Keelor; Assistant Secretary, S. Sisson; Librarian, W. Corlis; Treasurer, E. C.

Porter. The following papers have been read before the Society in the way of Essays and Communications during the month of February, and were well discussed by members present. Essays: "Breeding the Trotter," by D. C. Langford; "Physiology of Kidneys," by J. E. Foster; "Animal Intelligence," by H. F. Vulliamy; "Contagious Pleura Pneumonia," by W. Gray; "Anthrax," by W. R. Taylor; "Bacteria," by A. G. Hopkins, and "Ergotism," by J. G. Parslow. Communications: "Bursatte," by U. B. McCurdy; "Laminitis" by A. J. Morrison; "Castrating a Riding Horse," by A. K. Kellam; "Fracture of the Metacarpal Bone," by G. W. Moore; "Ovariotomy in a Heifer," by J. H. Honan; "Firing in Ring Bone," by J. A. Pendergast and "Fracture of the Os Pedis," by J. W. Rollins.

ALLEN Z. KEELOR, *Secretary.*

